# Initial stem cell adhesion on porous silicon surface: molecular architecture of actin cytoskeleton and filopodial growth

**DOI:** 10.1186/1556-276X-9-564

**Published:** 2014-10-10

**Authors:** Pierre-Yves Collart-Dutilleul, Ivan Panayotov, Emilie Secret, Frédérique Cunin, Csilla Gergely, Frédéric Cuisinier, Marta Martin

**Affiliations:** 1 BioNano Laboratory EA 4203, Université Montpellier 1, Montpellier 34193, France; 2 Matériaux Avancés Catalyse Santé, Institut Charles Gerhardt, UMR 5253 CNRS, Montpellier 34296, France; 3 Laboratoire Charles Coulomb UMR 5221, Université Montpellier 2, Montpellier 34095, France; 4 Laboratoire Charles Coulomb UMR 5221, CNRS, Montpellier 34095, France

**Keywords:** Mesenchymal stem cells, Cell adhesion, Porosity, Tissue engineering

## Abstract

The way cells explore their surrounding extracellular matrix (ECM) during development and migration is mediated by lamellipodia at their leading edge, acting as an actual motor pulling the cell forward. Lamellipodia are the primary area within the cell of actin microfilaments (filopodia) formation. In this work, we report on the use of porous silicon (pSi) scaffolds to mimic the ECM of mesenchymal stem cells from the dental pulp (DPSC) and breast cancer (MCF-7) cells. Our atomic force microscopy (AFM), fluorescence microscopy, and scanning electron microscopy (SEM) results show that pSi promoted the appearance of lateral filopodia protruding from the DPSC cell body and not only in the lamellipodia area. The formation of elongated lateral actin filaments suggests that pores provided the necessary anchorage points for protrusion growth. Although MCF-7 cells displayed a lower presence of organized actin network on both pSi and nonporous silicon, pSi stimulated the formation of extended cell protrusions.

## Background

In the field of regenerative medicine, tissue engineering offers promises for the treatment of various human diseases. Biologic tissues consist of cells, extracellular matrix (ECM), and signaling systems, activating genes or transcriptional factors responsible for tissue growth and differentiation. Thus, tissue engineering aims at recreating tissues that are defective or lost and is based on biological substitutes to repair physiological tissue functions by combining cells, bioactive factors, and biomaterial scaffolds [1]. Cell-based therapies are driven by the observation that tissue or organ transplantation brings significant benefits to treated patients. Cell therapies are not only processing cells or tissues but also engineering and manufacturing cell-based products. Cellular engineering procedures motivate the development of scaffolds incorporating cell-level parameters such as endogenously produced factors and favorable physicochemical microenvironment [2]. An essential challenge in tissue engineering is the understanding of cell-substrate interactions, which are involved in the difference observed in cell behavior when comparing in vitro and in vivo culturing [3,4]. Indeed, stem cell-based therapies often require a scaffold to carry stem cells to the injured site [5]. In human body, cells are in contact with each other and with ECM, all of them exhibiting nanostructures such as nanopores, nanofibers, or arrangements of adhesion proteins. Cell behavior is determined by intrinsic and extrinsic cell signals (from the surrounding cells and ECM). The extrinsic signals are of both chemical (bioactive molecules) and mechanical (forces caused by the cell-cell or cell-ECM interactions, at the micrometer and nanometer scale) nature [3]. The way cells explore their surrounding ECM during development and migration is mediated by protrusion of actin-rich structures at the cell front. These protrusive structures are commonly called lamellipodium, which are built of a dense network of actin filaments. Lamellipodium protrusions are frequently accompanied by the formation of bundles of parallel actin filaments, usually termed filopodia. Lamellipodia frequently embed actin filament microspikes. These microspikes can develop into filopodia by protruding beyond the lamellipodium edge, but also as peripheral actin filament retraction fibers, associated with the sides of migrating cells [6]. Filopodia are dynamic structures that rapidly extend and retract, found at the edge of various motile cells, as well as the growth cone tips of migrating axons. Cells use filopodia as sensor of the local environment to explore surfaces of other cells and surrounding ECM, in order to identify appropriate adhesion sites [7,8]. A central concern of tissue engineering is to understand how to control the microenvironment surrounding the cells to restore similar conditions for cells as in their innate environment. For this, micropatterning has been used to create substrates with controlled architecture and optimized surface chemical properties. The topography of the substrate surface appeared to exert an effect on cells independently to surface chemistry [4]. Thus, a biomaterial scaffold for tissue engineering should ideally mimic the chemical and mechanical properties of in vivo environment, in order to support cell attachment, proliferation, and differentiation. In this field, porous silicon (pSi) appears to be a promising biomaterial as it is both nontoxic and bioresorbable under physiological conditions and dissolves progressively into nontoxic silicic acid [5,9,10]. PSi is a semiconductor material obtained by electrochemical etching of flat silicon. Nanostructured pSi has been studied and used as a biomaterial with many advantages over existing alternatives as it is inorganic and can be readily sterilized. Pore dimensions can be controlled and tuned, from micropores (<2 nm) through mesopores (2 to 50 nm) to macropores (>50 nm up to several microns). It is well tolerated and noninflammatory within the body, and it has the ability to degrade completely in aqueous solutions into nontoxic silicic acid, the major form of silicon in the human body [11,12]. Depending on the chemical doping of the silicon material base, conditions of fabrication and the surface functionalization applied, the degradation kinetics can vary from hours to months [10,12]. And pSi has been shown to support the attachment and growth of a variety of mammalian cells [13-15]. Moreover, in physiological concentrations, it has been demonstrated that silicic acid stimulated collagen type I synthesis and osteoblast differentiation [16], highlighting the positive effect of Si on bone tissue healing. PSi structure has also been shown to favor calcium phosphate nucleation [17].

In vitro, interactions with the culture surface allow cells to respond to various mechanical forces, and surface porosity influences initial cell attachment and spreading, where cells attach to and pull on the matrix [18]. Cell spreading can be described as a balance between cellular forces of protrusion, contraction, and adhesion. The micro- and nanoarchitecture plays an important role by promoting or not cell attachment and spreading [19].

Stem cell biology is an important field for the understanding of tissue regeneration and further regenerative medicine. Mesenchymal stem cells (MSCs) have been isolated first from bone marrow and then from various tissues, such as adipose tissue, umbilical cord blood, dental pulp, and many others. MSCs are adherent fibroblast-like cells able to proliferate in vitro and capable of giving rise to at least three cell lineages: osteogenic, chondrogenic, and adipogenic. The first type of dental stem cell was isolated from the human pulp tissue and named dental pulp stem cells (DPSCs) [20]. They have the ability to differentiate into various cell lineages, such as osteogenic, adipogenic, chondrogenic, neurogenic, myogenic, dentogenic, and cementogenic [21]. Moreover, they are easily accessible after tooth extraction, especially wisdom teeth extraction for orthodontic reasons [22].

We aimed to study the influence of pSi porosity on cell adhesion, especially through lamellipodia and filopodia formation. The effect of pore size and porosity on cell growth is of particular relevance, as it will direct the fabrication of a porous biomaterial. Although microscale topography modulates cellular behavior in vitro, it is important to consider that cells in vivo make contact with nanoscale as well as microscale topographical features. Even if cells are typically tens of microns in diameter, the dimensions of subcellular structures tend to the nanometer scale [10,23]. Therefore, we considered the impact of pSi porosity on cell adhesion and spreading. We investigated the behavior of primary culture of human DPSC on pSi scaffolds with pore diameters ranging from 30 to 40 nm. We focused on the formation of actin network and protrusions according to the presence of pores, using atomic force microscopy (AFM), fluorescence microscopy, and scanning electron microscopy (SEM). Nonporous silicon substrate (flat Si) and breast cancer (MCF-7) cell lines were used as controls. MCF-7 cell lines are a standard model of metastatic cancer cells, adherent, proliferative, and capable of living several months in vitro. Unlike MSCs, they are not able to differentiate and have limited spreading capacities [24,25].

## Methods

### Porous silicon scaffolds

Porous silicon scaffolds were created from P^++^ type boron-doped crystalline silicon wafers with 0.0008 to 0.0012 Ω cm resistivity, obtained from Siltronix (Siltronix, Archamps, France). Wafers were etched in a custom-made Teflon cell at a constant current density of 300 mA/cm^2^ for 2 min 15 s, in a hydrofluoric acid (HF) solution in ethanol (3:1 HF/ethanol solution, volume ratio). Etched wafers were oxidized at 800°C for 1 h and cut into 0.5-cm^2^ pieces. Nonetched silicon wafers (flat Si) were also oxidized at 800°C for 1 h and used as control. To characterize pSi pore diameter, the topography of the surface was analyzed by environmental scanning electron microscopy (SEM) (Analytic FEI Quanta FEG 200, FEI, Hillsboro, OR, USA) with an acceleration voltage of 20.00 kV in a pressure of 0.5 Torr. SEM images were treated and analyzed using ImageJ® software to measure the mean pore diameter. For cell culture experiments, pSi and flat Si scaffolds were sterilized in 70% ethanol for 10 min, and cells were seeded onto the surface of sterilized pSi or flat Si at a density of 10^5^ cell/mL.

### Human dental pulp stem cells

Human impacted third molar extracted for orthodontic reasons were recovered from healthy patients (15 to 18 years of age), after written informed consent, following a protocol approved by the local ethical committee (Comité de Protection des Personnes, Montpellier hospital, France). Dental pulp cells were recovered after pulp enzymatic digestion in a solution of 3 mg/mL collagenase type I and 4 mg/mL dispase (BD Biosciences, Bedford, MA, USA). Cells were incubated for 1 week in 75-mL flasks, at 37°C with 5% CO_2_, in αMEM supplemented with 10% fetal bovine serum (Invitrogen, Carlsbad, CA, USA). Nonadherent cells were removed by a change of medium 24 h after cell seeding. After 1 week, subconfluent cells were collected and analyzed for minimal criteria to define human mesenchymal stem cells, such as adherence to plastic, expression of cell surface antigens, and ability to differentiate into osteoblasts, adipocytes, and chondroblasts in vitro [26].

### MCF-7

We used MCF-7 breast cancer cell lines as a control in our experiments. MCF-7 cell lines were obtained from the American Type Culture Collection (ATCC, Manassas, VA, USA). Cells were cultured at 37°C in a humidified atmosphere with 5% CO_2_, in DMEM containing 4.5 g/L d-Glucose, supplemented with 10% fetal bovine serum (all from Invitrogen, Carlsbad, CA, USA).

### Atomic force microscopy

An Asylum MFP-3D atomic force microscopy (AFM) system (Asylum Research, Santa Barbara, CA, USA), mounted on an Olympus inverted microscope (Olympus Corporation, Shinjuku-ku, Japan), was used for cell imaging. Triangular silicon nitride cantilevers (MLCT-AUHW, Veeco, Plainview, NY, USA) with a nominal spring constant of 10 pN/nm and half-opening angle of 40°, were used. The spring constant for each cantilever was determined by thermal noise method within the supplied software. Cells were examined after 24 h of in vitro incubation on pSi scaffolds or on flat Si. Cells were fixed in 2.5% glutaraldehyde for 1 h, then rinsed three times in phosphate buffer saline (PBS) (Invitrogen, Carlsbad, CA, USA). AFM topographic images were obtained using contact mode in liquid (PBS) at room temperature.

### Fluorescence microscopy

Cells were incubated for 24 h at 37°C with 5% CO_2_, in a humidified incubator, then fixed in 3.7% formaldehyde for 20 min at room temperature, and stained for actin cytoskeleton and nuclei. Cells were permeabilized with 0.5% Triton X-100 in PBS at 4°C for 15 min, incubated for 1 h with TRITC-labeled phalloidin (1:200) at 37°C in the dark, then with Hoechst 33342 (Life Technologies, Carlsbad, CA, USA) for 10 min at room temperature, and washed twice with deionized water. Samples were observed under fluorescence microscopy (Nikon TE2000, Nikon Instruments, Amsterdam, Netherlands) at an excitation wavelength of 360 nm for Hoechst staining (nucleus staining) and 630 nm for phalloidin (actin staining).

### Scanning electron microscopy

Cells were cultured on pSi and flat Si scaffolds for 24 h under normal conditions, as described above. After incubation, cells were washed with PBS and fixed with 2.5% glutaraldehyde for 1 h at room temperature. Samples were dehydrated in graded ethanol solutions from 50 to 100% and in hexamethyldisilazane (HMDS, Ted Pella, Redding, CA, USA), then sputter-coated with platinum. Scanning electron microscopy (SEM) was performed on an Analytic FEI Quanta FEG 200 microscope (FEI, Hillsboro, OR, USA) with an acceleration voltage of 15 kV in a pressure of 1 × 10^−5^ Torr.

## Results and discussion

### PSi surface characterization

**Figure 1:**
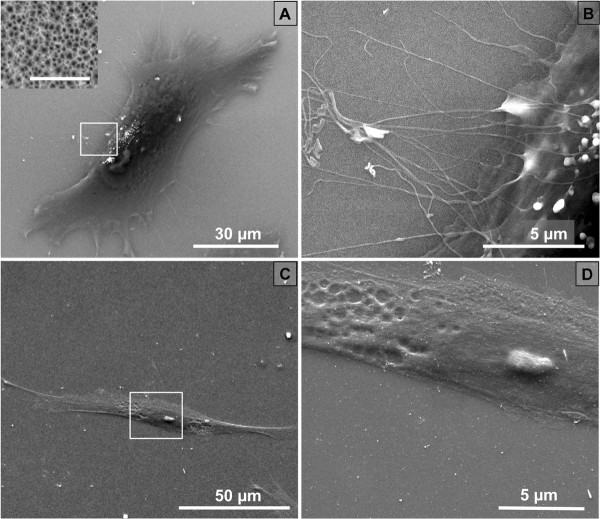
**Scanning electron microscopy of DPSCs. (A)** DPSC on pSi; magnification × 3,000, scale bar = 30 μm. Inset shows higher magnification of the surface (×150,000) with visible pores, scale bar = 500 nm. **(B)** Higher magnification (square in A) of DPSC on pSi with protrusions from the cellular body; magnification × 20,000, scale bar = 5 μm. **(C)** DPSC on flat Si; magnification × 2,000, scale bar = 50 μm. **(D)** Higher magnification (square in C) of DPSC on flat Si; magnification × 17,000, scale bar = 5 μm.

Porous substrates were obtained via anodization of p-type silicon wafers, in a constant current density of 300 mA/cm^2^, as previously described [5]. SEM examination of pSi surfaces revealed an average pore diameter of 36 ± 4 nm (inset, Figure 1A). Various chemical modifications of pSi surfaces to promote cell adhesion have been described [5,14]. In this study, we focused on the response of filopodia/lamellipodia to a porous surface at the nanometer scale independently of the chemical treatment; thereby, a simple thermal oxidation of the PSi surfaces was realized.

### Cell morphology by SEM observation

Cell morphology is responsive to topographical features of the substrate surface, and the way cells adhere and spread on the surface influences their shape, growth, and differentiation [27]. On both flat and porous surfaces, DPSCs spread widely with typical long axis size ranging from 60 to 80 μm and formed lamellipodia at the leading edge or even at both edges. SEM images of DPSCs on pSi and flat Si are presented in Figure 1.

**Figure 2:**
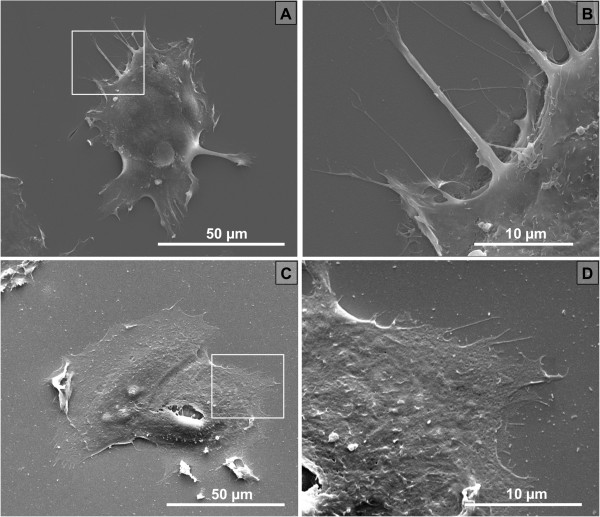
**Scanning electron microscopy of MCF-7. (A)** MCF-7 on pSi, magnification × 2,500. **(B)** Higher magnification (square in A) of MCF-7 on pSi with few protrusions from the cellular body, magnification × 10,000. **(C)** MCF-7 on flat Si, magnification × 3,000. **(D)** Higher magnification (square in C) of MCF-7 on flat Si, magnification × 10,000.

On flat Si, DPSCs developed short filopodia at their apical pole, spreading from lamellipodia, as usually observed for fibroblast-like cells [6,28,29]. On pSi samples, DPSC developed similar filopodia spreading from lamellipodia, but they also developed long and thin protrusions, growing directly from the cellular body, with a length of tens of micrometers (Figure 1B). The culture of MCF-7 breast cancer cell lines did not present clear development of lamellipodia, with no difference in terms of shape or spreading. However, some long and thin protrusions were observed on pSi while they were hardly visible on flat Si (Figure 2).

### Actin cytoskeleton observation

**Figure 3:**
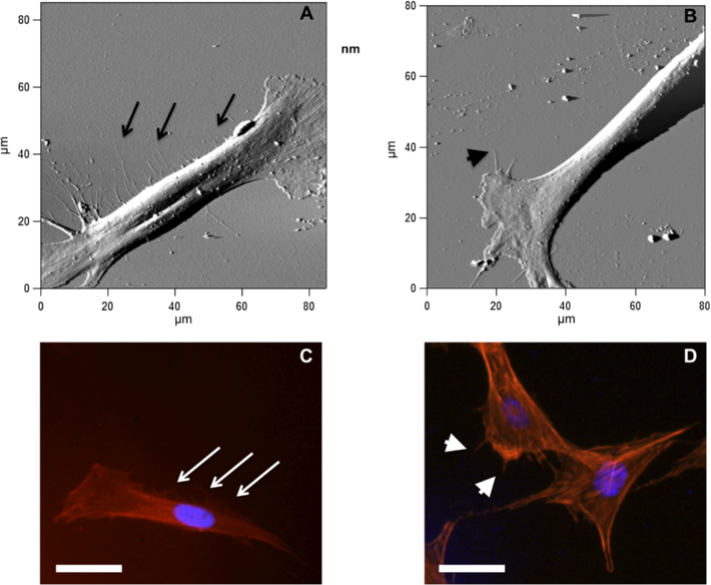
**Atomic force microscopy and fluorescence microscopy images of DPSCs on pSi and flat Si.** Representative AFM deflection images of DPSC on pSi **(A)** and flat Si **(B)**. Fluorescence microscopy images of DPSCs on pSi **(C)** and flat Si **(D)** with actin staining (red) and nuclei staining (blue), showing the filopodia formations. Scale bar = 20 μm. The arrows highlight the long thin filopodia growing from the main cell body, while the arrowheads show filopodia growing from lamellipodia.

We were interested in filopodia formation for DPSC growing on pSi samples, as this gives information about cell behavior since these cellular projections contribute to intercellular communication, cell adhesion, and motility [28]. Indeed, cell spreading and motility require the extension driven by the assembly of actin. Lamellipodia are formed by a dense network of cross-linked actin filaments, and filopodia are exploratory extensions formed by parallel bundles of actin filaments coming from the plasma membrane [6,29]. Moreover, surface topography can affect the extension and adhesion of filopodia and lamellipodia [30-33]. Therefore, we investigated DPSC adhesion using fluorescence after actin and nuclei staining. Fluorescence microscopy images are presented in Figure 3. The long and thin lateral protrusions observed for DPSCs on pSi were formed by actin, confirming them as filopodia. Indeed, the actin structures, which play fundamental roles in cell migration, can be separated into three main categories: lamellipodial actin network at the leading edge of the cell, unipolar filopodial bundles beneath the plasma membrane, and contractile actin stress fibers in the cytoplasm [34]. Filopodia are the only actin structure protruding from the plasma membrane. Figure 3 presents a comparison between AFM images and fluorescence images, where lamellipodia and related filopodia are clearly visible on flat Si and pSi, while lateral filopodia are observed only on pSi (Figure 3A). These experiments were repeated with MCF-7 breast cancer cell lines, used as control. We indeed observed a lower presence of organized actin network or bundles, on both surfaces (Figure S1 in Additional file 1).

### Filopodia characterization by AFM

**Figure 4:**
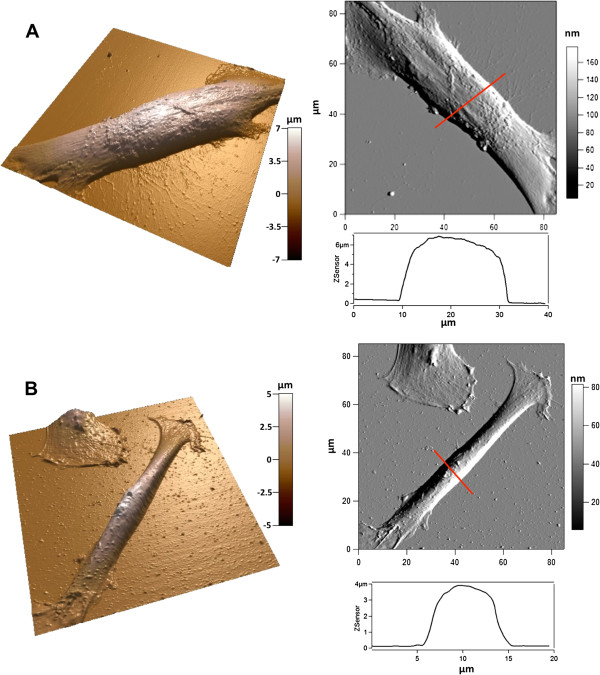
**AFM morphology study of DPSCs.** DPSCs on pSi **(A)** and flat Si **(B)**. Images show 3D reconstructions of AFM height images (not shown) with the corresponding deflection images and height profiles of the cell body along the indicated red lines.

**Figure 5:**
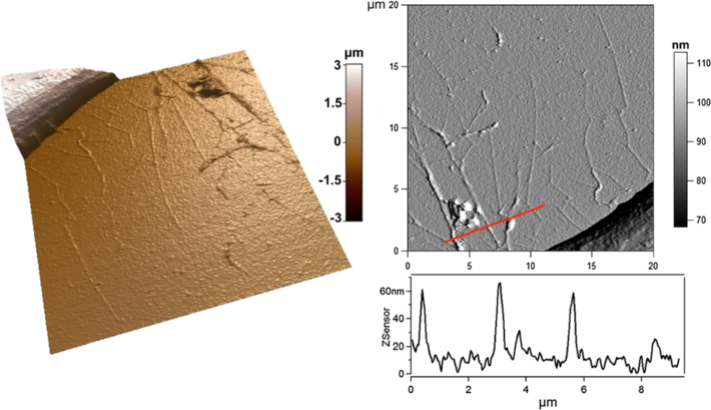
**AFM images in contact mode of a DPSC cultured on pSi.** 3D reconstruction of the AFM height image of the side of a cell, with the corresponding deflection image and height cross section along the indicated red line.

We investigated cell spreading and filopodia formation by AFM. Representative 3D images and deflection images are shown in Figure 4. On both flat Si and pSi, DPSCs formed well-organized lamellipodia at their leading edge, with short filopodial protrusions growing from these areas. We also confirmed that long and thin filopodia were growing from the main cell body on pSi surfaces, in addition to those protruding from lamellipodia (Figure 4A). Cross section measurements of 45 filopodia protruding from 10 DPSCs on pSi and 8 filopodia from 3 DPSCs on flat Si were performed, yielding to an average height of 67 ± 34 nm and width of 418 ± 269 nm for the former and an average height of 46 ± 16 nm and width of 206 ± 33 nm for the latter. Our results indicate that protrusions from DPSCs on pSi are higher and wider than the ones from DPSCs on flat Si, evidencing a rather high dispersion from the average value. Indeed, the analyzed filopodia presented a wide range of lengths, suggesting different stages of growth. Some cross sections are presented in Figure 5. In mesenchymal migration, the cells attach to and pull on the matrix, through focal adhesion and cytoskeleton. Filopodia act as cellular tentacles to bind to a surface then retract and pull the cell toward the bound surface. In addition, it has been shown that actin-dependent retraction of the filopodia occurred with discrete steps, with an average step size of 36 ± 13 nm [8].

**Figure 6:**
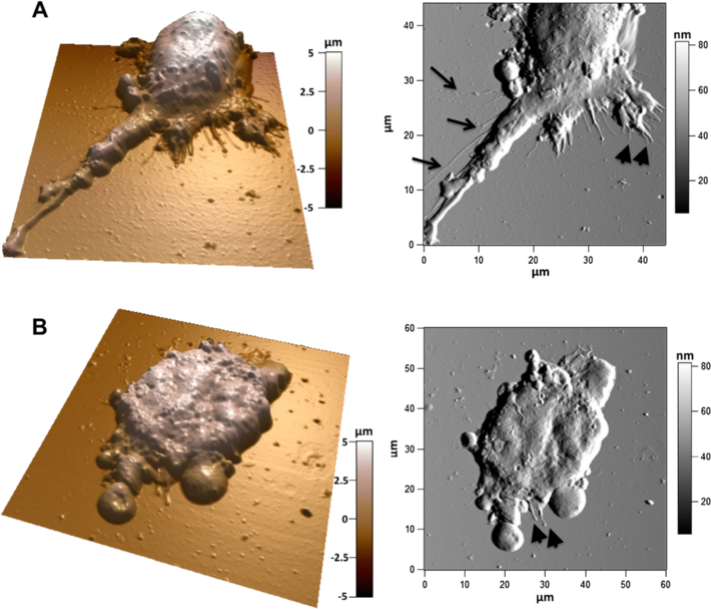
**AFM morphology study of MCF-7.** MCF-7 on pSi **(A)** and flat Si **(B)**. Images show 3D reconstructions of AFM height images (not shown) with the corresponding deflection images. The arrows highlight the long thin filopodia growing from the main cell body, while the arrowheads show filopodia growing from lamellipodia.

**Figure 7:**
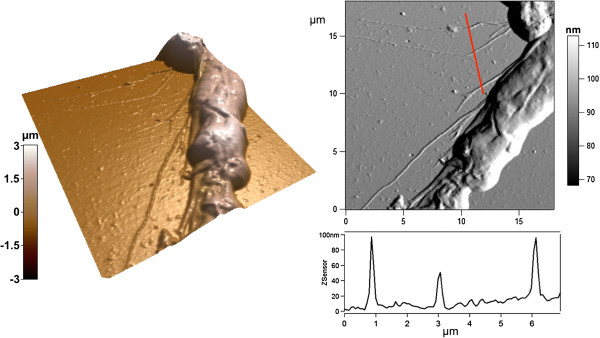
**AFM images in contact mode of a MCF-7 cultured on pSi.** 3D reconstruction of the AFM height image of the side of a cell, with the corresponding deflection image and height cross section along the indicated red line.

Experiments were repeated with MCF-7. We confirmed that cells were able to extend protrusions only on the porous surfaces, even though these protrusions were fewer compared to DPSCs (Figure 6). We determined the size of the protrusions by cross section analysis. Some examples are presented in Figure 7. Cross section measurements of 11 filopodia protruding from 3 MCF-7 cells on pSi and 8 filopodia from 4 MCF-7 cells on flat Si were performed, giving an average height of 36 ± 18 nm and width of 410 ± 123 nm for the former and an average height of 55 ± 20 nm and width of 145 ± 67 nm for the latter. These sizes are roughly in the same range as those of the filopodia observed with DPSCs and indicate that protrusions from MCF-7 cells on pSi are much wider than the ones from MCF-7 cells on flat Si. The results also present a high standard deviation. Furthermore, we studied the density of filopodia for DPSC and MCF-7 on pSi and flat Si by means of SEM. A synthesis of the main results is presented in Table S1 in Additional file 2.

The lateral filopodia were able to extend only on the porous surface leading to the hypothesis that pores provided anchorage points for protrusion growth and possible further cell spreading and crawling. The directional extension of lamellipodia on both surfaces modulated cell morphology, resulting in a fibroblast-like morphology. It has already been shown that cytoskeletal tension was related to the surface topography of substrates, with filopodia detecting surface topography, converting into lamellipodia and directing cell spreading and shape [35,36]. DPSCs are multipotent stem cells able to give rise to multilineage differentiation [20,21,37,38]. The investigation of the relationship between filopodia/lamellipodia formations, cytoskeletal tension, and surface nanopatterning aims to elucidate the impact of surface topography on cell spreading, migration, and further differentiation.

### Role of filopodia on initial cell response to pSi surface

Filopodia have been shown to play an important role in cell migration: these thin actin protrusions can probe the extracellular environment and guide cell migration toward sites of interest. They can also be involved in other cellular processes such as wound healing, adhesion to the extracellular matrix, guidance toward chemoattractants, and neuronal growth-cone pathfinding [28]. Filopodia are linked to the enhancement of directed cell migration, because they are essential components, with lamellipodia, for cell motility. This correlates with the role of filopodia in sensing the cell's surroundings and acting as sites for signal transduction [29]. We showed in this study that porous surface had a strong impact on filopodia formation, as lateral actin protrusions were observed only on pSi and not on flat Si. Previous studies enlightened the influence of pSi nanoscale textured micropatterns on mesenchymal stem cell adhesion and migration, with cells migrating toward the porous areas [39]. According to the pattern size, cellular filopodia can even bypass some unfavorable areas to reach some more suitable surfaces [40]. The mechanisms underlying cell-topography interactions remain elusive but have a major impact on cell spreading and migration. These interactions concern not only porous structure but also various surface topographies. Similar results have recently been presented on microsphere array patterns promoting MSC adhesion and spreading through filopodia formation and conversion of filopodia into lamellipodia [27].

No organized actin structures were observed with MCF-7 on pSi and flat Si. These results are in accordance with previous published works, showing that actin structures in MCF-7 formed a disorganized network with few filaments at the basal plane of the cell [41]. The presence of reduced organized actin filaments in MCF-7 was observed in this work as well and used as a negative control. However, some elongated thin protrusions growing from MCF-7 could be observed when the cells were incubated on pSi.

Cell-scaffold interactions play a significant role in tissue engineering to guide cell spreading and migration. Even if the mechanisms underlying these interactions remain elusive, we assume pSi porosity at the nanometer scale promotes environment exploration through filopodia formation and further cell migration. Filopodia can turn into lamellipodia to initiate the directional formation and extension of lamellipodia. Filopodia are not only acting as probes to detect microenvironment but also serve as skeleton to guide lamellipodia extension for directing cell motions, in response to surface topography [27]. In our findings, it remains elusive whether filopodia appeared first and some of them guided the formation of lamellipodia or if lateral filopodia were probing microenvironment during cell migration toward the leading edge (where the lamellipodia is). Here, we show that filopodia contain a core of extending actin filament bundles. We assume that cell actively probes the external environment on pSi surface to gather spatial, topographical, and chemical information from the material surface. Such hair-like filopodial protrusions have been thought to locate specific ECM protein motifs and initiate integrin receptor clustering and focal adhesion protein recruitment, which is the first step of mature adhesion formation, before potential stem cell differentiation [23].

## Conclusions

PSi is indeed a promising nanostructured biomaterial scaffold to be easily used in tissue engineering as it ideally mimics the ECM environment properties, in order to support cell attachment, development, and migration. Our work demonstrates that pSi plays an important role in promoting the formation of lateral actin microfilaments protruding from the DPSC cell body and from the lamellipodia area. Here, pSi porosity seems to provide the necessary anchorage points for filopodia growth. The observed lateral filopodia could act as supplementary probes to explore the surrounding environment. Moreover, control experiments showed that pSi permits the development of filopodia-like structures, even with MCF-7 cancerous cell lines. We believe these results might be of importance for tissue engineering applications. Further work is required and is currently on process to assess the impact of porosity on anchorage protein organization in vitro and the effect in vivo of such a biocomplex MSC-pSi.

## Competing interests

The authors declare that they have no competing interests.

## Authors’ contributions

The experiments presented in this work were designed by FCi, MM, CG, and PYCD. The pSi fabrication by electrochemical etching was done by ES and FCn. PYCD, ES, FCn, and MM participated in pSi surface characterization. Cell experiments with fluorescence microscopy and SEM were performed by PYCD and IP. MM and PYCD studied the filopodia formation by AFM. The manuscript was written by PYCD, MM, FCn, CG, and FCi, and the last version was revised by all the authors. All authors read and approved the final manuscript.

## Supplementary Material

Additional file 1: Figure S1 Fluorescence microscopy images of MCF-7 and DPSC on pSi and flat Si, with actin staining (red) and nuclei staining (blue). MCF-7 on pSi (A) and flat Si (B). DPSC on pSi (C) and flat Si (D). Scale bar = 30 μm. Click here for file

Additional file 2: Table S1 Presence of filopodia for DPSC and MCF-7 on pSi and flat Si. Click here for file

## References

[B1] Bell E Lanza RP, Langer R, Vacanti J Tissue engineering in perspective Principles of Tissue Engineering 2000 San Diego, USA: Academic 3 7

[B2] Kirouac DC Zandstra PW The systematic production of cells for cell therapies Cell Stem Cell 2008 3 369 381 10.1016/j.stem.2008.09.001 18940729

[B3] Martinez E Engel E Planell JA Samitier J Effects of artificial micro- and nano-structured surfaces on cell behaviour Ann Anat 2009 191 126 135 10.1016/j.aanat.2008.05.006 18692370

[B4] Yim EKF Leong KW Significance of synthetic nanostructures in dictating cellular response Nanomedicine 2005 1 10 21 10.1016/j.nano.2004.11.008 17292053

[B5] Collart Dutilleul P-Y Secret E Panayotov I Deville de Périère D Martin-Palma RJ Torres-Costa V Martin M Gergely C Durand J-O Cunin F Cuisinier F Adhesion and proliferation of human mesenchymal stem cells from dental pulp on porous silicon scaffolds ACS Appl Mater Interfaces 2014 6 1719 1728 10.1021/am4046316 24428409

[B6] Faix J Rottner K The making of filopodia Curr Opin Cell Biol 2006 18 18 25 10.1016/j.ceb.2005.11.002 16337369

[B7] Wood W Martin P Structures in focus—filopodia Int J Biochem Cell Biol 2002 34 726 730 10.1016/S1357-2725(01)00172-8 11950590

[B8] Kress H Stelzer EHK Holzer D Buss F Griffiths G Rohrbach A Filopodia act as phagocytic tentacles and pull with discrete steps and a load-dependent velocity Proc Natl Acad Sci U S A 2007 104 11633 11638 10.1073/pnas.0702449104 17620618 PMC1913848

[B9] Park J-H Gu L von Maltzahn G Ruoslahti E Bhatia SN Sailor MJ Biodegradable luminescent porous silicon nanoparticles for in vivo applications Nat Mater 2009 8 331 336 10.1038/nmat2398 19234444 PMC3058936

[B10] Collart Dutilleul P-Y Deville de Périère D Cuisinier FJ Gergely C Cunin F Santos H Porous silicon scaffolds for stem cells growth And osteodifferentiation Porous Silicon for Biomedical Applications 2014 Cambridge, UK: Woodhead Pub Limited 486 506

[B11] Canham LT Bioactive silicon structure fabrication through nanoetching techniques Adv Mater 1995 7 1033 1037 10.1002/adma.19950071215

[B12] Low SP Voelcker NH Canham LT Williams KA The biocompatibility of porous silicon in tissues of the eye Biomaterials 2009 30 2873 2880 10.1016/j.biomaterials.2009.02.008 19251317

[B13] Sapelkin AV Bayliss SC Unal B Charalambou A Interaction of B50 rat hippocampal cells with stain-etched porous silicon Biomaterials 2006 27 842 846 10.1016/j.biomaterials.2005.06.023 16098578

[B14] Low SP Williams KA Canham LT Voelcker NH Evaluation of mammalian cell adhesion on surface-modified porous silicon Biomaterials 2006 27 4538 4546 10.1016/j.biomaterials.2006.04.015 16707158

[B15] Alvarez SD Derfus AM Schwartz MP Bhatia SN Sailor MJ The compatibility of hepatocytes with chemically modified porous silicon with reference to in vitro biosensors Biomaterials 2009 30 26 34 10.1016/j.biomaterials.2008.09.005 18845334 PMC2784167

[B16] Hing KA Revell PA Smith N Buckland T Effect of silicon level on rate, quality and progression of bone healing within silicate-substituted porous hydroxyapatite scaffolds Biomaterials 2006 27 5014 5026 10.1016/j.biomaterials.2006.05.039 16790272

[B17] Canham LT Reeves CL Loni A Houlton MR Newey JP Simons AJ Cox TI Calcium phosphate nucleation on porous silicon: factors influencing kinetics in acellular simulated body fluids Thin Solid Films 1997 297 304 307 10.1016/S0040-6090(96)09534-X

[B18] Miron-Mendoza M Seemann J Grinnell F The differential regulation of cell motile activity through matrix stiffness and porosity in three dimensional collagen matrices Biomaterials 2010 31 6425 6435 10.1016/j.biomaterials.2010.04.064 20537378 PMC2900504

[B19] Haridas V Sadanandan S Collart Dutilleul P-Y Gronthos S Voelcker NH Lysine-appended polydiacetylenes scaffolds for human mesenchymal stem cells Biomacromolecules 2013 15 582 590 24364714 10.1021/bm4015655

[B20] Gronthos S Mankani M Brahim J Robey PG Shi S Postnatal human dental pulp stem cells (DPSCs) in vitro and in vivo Proc Natl Acad Sci U S A 2000 97 13625 13630 10.1073/pnas.240309797 11087820 PMC17626

[B21] Huang GT-J Gronthos S Shi S Mesenchymal stem cells derived from dental tissues vs. those from other sources: their biology and role in regenerative medicine J Dent Res 2009 88 792 806 10.1177/0022034509340867 19767575 PMC2830488

[B22] Atari M Gil-Recio C Fabregat M García-Fernández D Barajas M Carrasco MA Jung H-S Alfaro FH Casals N Prosper F Ferrés-Padró E Giner L Dental pulp of the third molar: a new source of pluripotent-like stem cells J Cell Sci 2012 125 3343 3356 10.1242/jcs.096537 22467856

[B23] Biggs MJP Richards RG Dalby MJ Nanotopographical modification: a regulator of cellular function through focal adhesions Nanomedicine 2010 6 619 633 10.1016/j.nano.2010.01.009 20138244 PMC2965469

[B24] Levenson AS Jordan VC MCF-7: the first hormone-responsive breast cancer cell line Cancer Res 1997 57 3071 3078 9242427

[B25] Salehi H Middendorp E Panayotov I Collart Dutilleul P-Y Dutilleul P-YC Vegh A-G Ramakrishnan S Gergely C Cuisinier F Confocal Raman data analysis enables identifying apoptosis of MCF-7 cells caused by anticancer drug paclitaxel J Biomed Opt 2013 18 56010 10.1117/1.JBO.18.5.056010 23698321

[B26] Dominici M Le Blanc K Mueller I Slaper-Cortenbach I Marini F Krause D Deans R Keating A Prockop D Horwitz E Minimal criteria for defining multipotent mesenchymal stromal cells Int Soc Cell Ther Position Statement Cytotherapy 2006 8 315 317 10.1080/14653240600855905 16923606

[B27] You R Li X Liu Y Liu G Lu S Li M Response of filopodia and lamellipodia to surface topography on micropatterned silk fibroin films J Biomed Mater Res A 2014 in press 10.1002/jbm.a.35097 24464986

[B28] Mattila PK Lappalainen P Filopodia: molecular architecture and cellular functions Nat Rev Mol Cell Biol 2008 9 446 454 10.1038/nrm2406 18464790

[B29] Small JV Stradal T Vignal E Rottner K The lamellipodium: where motility begins Trends Cell Biol 2002 12 112 120 10.1016/S0962-8924(01)02237-1 11859023

[B30] Parker KK Brock AL Brangwynne C Mannix RJ Wang N Ostuni E Geisse NA Adams JC Whitesides GM Ingber DE Directional control of lamellipodia extension by constraining cell shape and orienting cell tractional forces FASEB J 2002 16 1195 1204 10.1096/fj.02-0038com 12153987

[B31] Brock A Chang E Ho C-C LeDuc P Jiang X Whitesides GM Ingber DE Geometric determinants of directional cell motility revealed using microcontact printing Langmuir 2003 19 1611 1617 10.1021/la026394k 14674434

[B32] Crouch AS Miller D Luebke KJ Hu W Correlation of anisotropic cell behaviors with topographic aspect ratio Biomaterials 2009 30 1560 1567 10.1016/j.biomaterials.2008.11.041 19118891

[B33] Bucaro MA Vasquez Y Hatton BD Aizenberg J Fine-tuning the degree of stem cell polarization and alignment on ordered arrays of high-aspect-ratio nanopillars ACS Nano 2012 6 6222 6230 10.1021/nn301654e 22717194

[B34] Hotulainen P Lappalainen P Stress fibers are generated by two distinct actin assembly mechanisms in motile cells J Cell Biol 2006 173 383 394 10.1083/jcb.200511093 16651381 PMC2063839

[B35] Fletcher DA Mullins RD Cell mechanics and the cytoskeleton Nature 2010 463 485 492 10.1038/nature08908 20110992 PMC2851742

[B36] Dalby MJ Gadegaard N Tare R Andar A Riehle MO Herzyk P Wilkinson CDW Oreffo ROC The control of human mesenchymal cell differentiation using nanoscale symmetry and disorder Nat Mater 2007 6 997 1003 10.1038/nmat2013 17891143

[B37] Gronthos S Brahim J Li W Fisher LW Cherman N Boyde A DenBesten P Robey PG Shi S Stem cell properties of human dental pulp stem cells J Dent Res 2002 81 531 535 10.1177/154405910208100806 12147742

[B38] D'Aquino R Graziano A Sampaolesi M Laino G Pirozzi G De Rosa A Papaccio G Human postnatal dental pulp cells co-differentiate into osteoblasts and endotheliocytes: a pivotal synergy leading to adult bone tissue formation Cell Death Differ 2007 14 1162 1171 10.1038/sj.cdd.4402121 17347663

[B39] Punzón-Quijorna E Sánchez-Vaquero V Muñoz-Noval Á Perez-Roldan MJ Martín-Palma RJ Rossi F Climent-Font A Manso-Silván M Garcia-Ruiz JP Torres-Costa V Nanostructured porous silicon micropatterns as a tool for substrate-conditioned cell research Nanoscale Res Lett 2012 7 396 10.1186/1556-276X-7-396 22799489 PMC3458952

[B40] Kaivosoja E Myllymaa S Kouri V-P Myllymaa K Lappalainen R Konttinen YT Enhancement of silicon using micro-patterned surfaces of thin films Eur Cell Mater 2010 19 147 157 20379964 10.22203/ecm.v019a15

[B41] Li QS Lee GYH Ong CN Lim CT AFM indentation study of breast cancer cells Biochem Biophys Res Commun 2008 374 609 613 10.1016/j.bbrc.2008.07.078 18656442

