# Supplementary figures and images for: Initial stem cell adhesion on porous silicon surface: molecular architecture of actin cytoskeleton and filopodial growth
Source: Nanoscale Res Lett. 2014 Oct 10;9(1):564. doi: 10.1186/1556-276X-9-564 (PMC4217708; doi:10.1186/1556-276X-9-564)

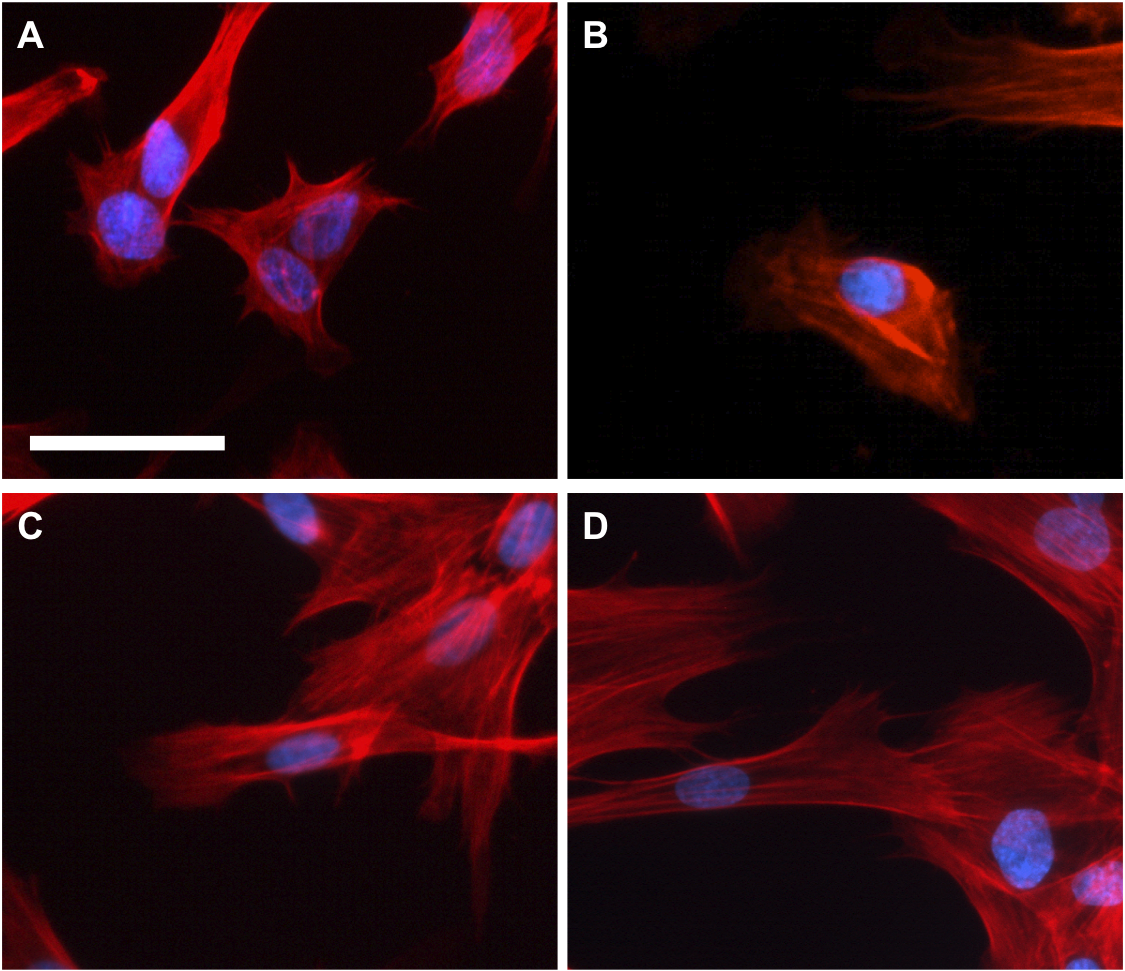

Supplement: Additional file 1: Figure S1 — Fluorescence microscopy images of MCF-7 and DPSC on pSi and flat Si, with actin staining (red) and nuclei staining (blue). MCF-7 on pSi (A) and flat Si (B). DPSC on pSi (C) and flat Si (D). Scale bar = 30 μm. [file 1556-276X-9-564-S1.tiff]
