# Supplementary material for: Initial stem cell adhesion on porous silicon surface: molecular architecture of actin cytoskeleton and filopodial growth
Source: Nanoscale Res Lett. 2014 Oct 10;9(1):564. doi: 10.1186/1556-276X-9-564 (PMC4217708; doi:10.1186/1556-276X-9-564)
Supplement: Additional file 2: Table S1 — Presence of filopodia for DPSC and MCF-7 on pSi and flat Si. [file 1556-276X-9-564-S2.doc]

Additional file 2: Table S1: Presence of filopodia for DPSC and MCF-7 on pSi and flat Si

|  | **DPSC pSi** | **DPSC flat Si** | **MCF-7 pSi** | **MCF-7 flat Si** |
| --- | --- | --- | --- | --- |
| **Number of Filopodia / Cell** | 47 ± 13 | 20 ± 9 | 29 ± 11 | 20 ± 4 |
| **Filopodia from Cell Body** | **+++** | **-** | **+** | **-** |
| **Filopodia from Lamellipodia** | **+** | **+** | **+** | **+** |
